# Transcriptome Changes Induced by Different Potassium Levels in Banana Roots

**DOI:** 10.3390/plants9010011

**Published:** 2019-12-19

**Authors:** Yingdui He, Ruimei Li, Fei Lin, Ying Xiong, Lixia Wang, Bizun Wang, Jianchun Guo, Chengxiao Hu

**Affiliations:** 1 College of Resource and Environment, Huazhong Agricultural University, Wuhan 430070, China; heyd@catas.cn; 2 Haikou Experimental Station, Chinese Academy of Tropical Agricultural Sciences, Haikou 571101, China; linfei198201@catas.cn (F.L.); xiongying950125@163.com (Y.X.); wlxmm@catas.cn (L.W.); Wangbizun168@catas.cn (B.W.); 3 Institute of Tropical Bioscience and Biotechnology, Chinese Academy of Tropical Agricultural Sciences, Haikou 571101, China; liruimei@itbb.org.cn; 4 College of Tropical Crops, Hainan University, Haikou 570228, China

**Keywords:** potassium, banana, transcriptome, root

## Abstract

Potassium plays an important role in enhancing plant resistance to biological and abiotic stresses and improving fruit quality. To study the effect of potassium nutrient levels on banana root growth and its regulation mechanism, four potassium concentrations were designed to treat banana roots from no potassium to high potassium. The results indicated that K2 (3 mmol/L K_2_SO_4_) treatment was a relatively normal potassium concentration for the growth of banana root, and too high or too low potassium concentration was not conducive to the growth of banana root. By comparing the transcriptome data in each treatment in pairs, 4454 differentially expressed genes were obtained. There were obvious differences in gene function enrichment in root systems treated with different concentrations of potassium. Six significant expression profiles (profile 0, 1, 2, 7, 9 and 13) were identified by STEM analysis. The hub genes were *FKF1, HsP70-1, NRT1/PTR5, CRY1*, and *ZIP11* in the profile 0; *CYP51* in profile 1; *SOS1* in profile 7; *THA, LKR/SDH, MCC, C4H, CHI, F3′H, 2 PR1s, BSP, TLP, ICS, RO*, chitinase and peroxidase in profile 9. Our results provide a comprehensive and systematic analysis of the gene regulation network in banana roots under different potassium stress.

## 1. Introduction

Potassium is a highly mobile nutrient mass that is an integral part of many physiological and biochemical processes in plants [1]. Potassium is essential for many cell and tissue activities, such as osmoregulation, enzyme activity, transport of minerals and metabolites, and stomatal aperture regulation [2,3,4,5,6,7]. It is involved in programmed cell death and senescence [8,9]. It also plays important roles in increasing plant resistance to disease [10,11,12,13]. Potassium deficiency can reduce plant photosynthesis, transpiration rate and stomatal conductance, and affect crop growth, yield quality and resistance to biological and abiotic stresses [7,14]. When plants face nutrient starvation in the soil, they will adjust the structure of root system and use the remaining resources to find a more favorable soil environment [15]. *Arabidopsis thaliana* significantly reduced lateral root elongation under low potassium treatment [16,17]. Maize varieties with high potassium uptake rate had advantages over those with low potassium uptake rate in terms of root length, root volume and root surface area [18]. The lack of potassium had a significant effect on the growth of rapeseed, and the lack of potassium significantly inhibited the growth of taproots and lateral roots [19]. Potassium deficiency of tobacco decreased root growth and mainly affected the formation and elongation of primary lateral roots [20]. Low potassium not only significantly reduced the dry weight of cabbage root, but also reduced its leaf area [21]. Therefore, the morphological structure of root system was greatly affected by potassium stress. It is significant to study the response mechanism of root system under potassium stress. There has been extensive research on root structural changes in response to stress of nutrients such as phosphate and nitrate and the signaling pathways that mediate these changes [22], and practical breakthroughs have been made. However, the mechanism of root structural change and response to potassium stress is still unclear.

With the purpose to explore the molecular mechanism of plant root response to potassium pressure, there are more and more reports on transcriptome, metabolome and proteomics of potassium stress (especially low potassium stress) in recent years. Transcriptome responses to K starvation in *Arabidopsis thaliana* [16], rice [4], soybean [23] and wild barley [24] indicated that genes related to ion transport, metabolism, signal transduction and protein phosphorylation significantly changed. Transcriptional analysis of sugarcane under low potassium stress showed significant differences in the expression of transcription factors, ion transporters, protein kinases and genes related to oxidative stress in the Ca^2+^ signal and ethylene signal pathways [25]. The results of transcriptome of rice potassium deficient seedlings showed that gene expression involved in nutrient transport, protein kinase, transcription process and plant hormone was significantly changed in roots [26]. Metabolomics analysis of tomato showed that potassium deficiency led to the soluble amino acids and soluble sugars increase in roots, meanwhile the organic acids and amino acids decrease in leaves [27]. ITRAQ proteome assessment showed that the differentially expressed proteins in wheat under low potassium stress showed organ-specific differences. In addition, most of the differently expressed proteins associated with hormone synthesis were found to be involved in JA synthesis [28].

Banana (*Musa acuminata* L.) is a kind of large herbaceous tropical and subtropical fruit tree. It is considered to be a potassium-loving fruit tree. In production, a large amount of potash fertilizer is often applied to increase banana yield, improve fruit quality and storage, and enhance plant resilience. However, due to the constant erosion of soil by seasonal rain, potassium was largely lost through leaching, and the use rate of potash fertilizer was less than 30% [29]. Excessive application of fertilizer not only causes waste, but also causes environmental pollution. Therefore, to explore the mechanism and regulation of banana response to low potassium stress is a feasible way to improve the use efficiency of banana potassium fertilizer and improve the efficiency of banana industry. In this study, “Brazilian” banana, the major variety planted in China, was used as an experimental subject. RNA-seq technology was applied on the roots to reveal the molecular mechanism of root growth affected by different potassium concentrations. Some important hub genes associated with ion transport, cell wall modification, transcript factors, hormone signaling were identified. This study provides an idea for the analysis of the molecular mechanism of potassium ion regulating banana root growth.

## 2. Results

### 2.1. Morphology Changes of Banana Roots after Potassium Treatments

**Figure 1:**
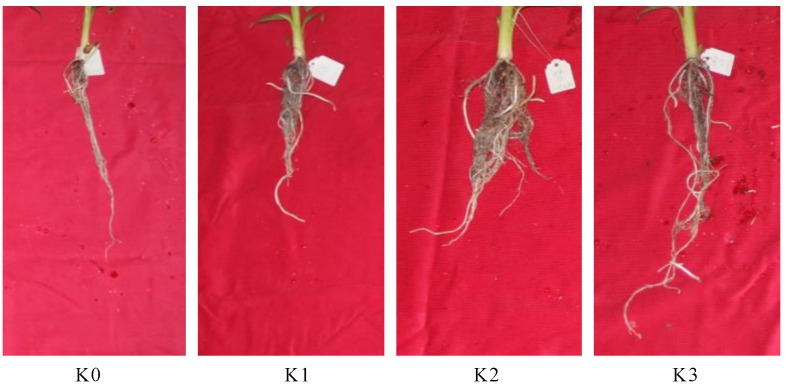
Effects of different concentration of potassium stress on the growth of banana roots. **K0**: 0 mmol/L K_2_SO_4_ treated banana root; **K1**: 0.03 mmol/L K_2_SO_4_ treated banana root; **K2**: 3 mmol/L K_2_SO_4_ treated banana root; **K3**: 200 mmol/L K_2_SO_4_ treated banana root.

Banana roots exposure to different concentration of potassium showed a significant different growth phenotype. The roots that treated with K2 (3 mmol/L K_2_SO_4_) concentration grew best, with strongest root and most root hairs (Figure 1). From K0 (0 mmol/L K_2_SO_4_) to K2, the total length of the roots does not change much, but the number of roots and root hairs increased with the potassium concentration. However, feed with the high level of potassium (K3, 200 mmol/L K_2_SO_4_), the roots got the longest root length, but less root numbers and root hairs (Figure 1). These results indicated that both too low or too high potassium concentrations inhibit the growth of banana roots, but in different ways.

### 2.2. Overview of Transcriptome Sequencing Results

To overview the banana roots gene expression patterns at different potassium supply conditions, we carried out the RNA-seq analysis. Seedling root samples treated as K0, K1, K2 and K3, were sequenced. Approximately 24.02 to 34.47 million 150 bp paired-end clean reads were obtained from K0, K1, K2 and K3 samples, respectively. The average Q20 contents were 94.01% to 94.15%, and GC contents were 53.58% to 54.26%, respectively. These data indicate that the sequencing results were fine. The mapped reads information among K0, K1, K2 and K3 samples was: 77.27% to 83.37% of the total mapping ratio (Table 1). The transcriptome data was qualified and considered appropriate for subsequent analysis.

### 2.3. Different Concentration of Potassium Stresses Affect Genes Expression in Banana Roots

**Figure 2:**
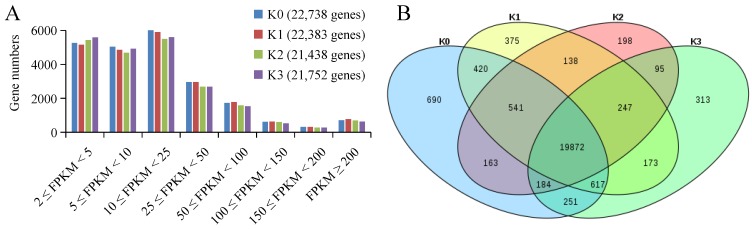
Transcript abundance measurements at each potassium concentration treatment. (**A**) The frequency represents the number of genes per category according to FPKM expression value. The number of total considered expressed genes (FPKM ≥ 2) for each moment is presented in brackets. (**B**) Venn diagram of expressed genes (FPKM ≥ 2) for each library (K0: 22,738 genes; K1: 22,383 genes; K2: 21,438 genes; K3: 21,752 genes). The number of common expressed genes in each intersection area is presented.

More than 68% of the genes had gene expression levels of FPKM ≥ 2 in each evaluated instance. Among the above gene clusters, more than 70% of genes were FPKM < 25, and more than 25% had FPKM between 10 and 25 (Table S1). About 3% of genes had FPKM values more than 200. It is clear that the gene expression levels in the four treatments displayed the same trend (Figure 2A). The gene expression distribution analysis showed that among the 24,277 genes with FPKM ≥ 2, 19,872 (82%) genes were expressed in all four potassium treatments, while a small number were expressed only in one condition (2.8% of K0, 1.5% of K1, 0.8% of K2, and 1.3% of K3) (Figure 2B).

**Figure 3:**
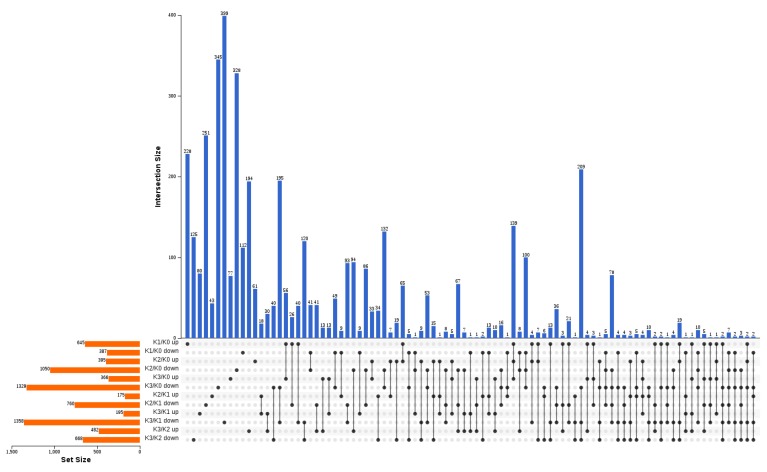
Upset Venn illustrating the number of DEGs between different concentration potassium treated banana roots. The horizontal orange bar graph on the left side represents the element statistics of each set, the single black point in the middle matrix represents the element unique to a set, the line between points and points represents the intersection unique to different sets, and the vertical blue bar graph represents the corresponding intersection element values.

By comparing each treatment in pairs, 4454 differently expressed genes (DEGs) were obtained (Figure 3). Compared to K0, 1032 (645 up-regulated, 387 down-regulated), 1445 (395 up-regulated, 1050 down-regulated), and 1695 (366 up-regulated, 1329 down-regulated) DEGs were detected after K1, K2 and K3 treatments, respectively (Figure 3). Compared to K1, a total of 935 (175 up-regulated, 760 down-regulated), and 1553 (195 up-regulated, 1358 down-regulated) DEGs were detected after K2 and K3 treatments, respectively (Figure 3). Compared to K2, 1050 (482 up-regulated, 668 down-regulated) DEGs were found after K3 treatment (Figure 3).

### 2.4. Gene function Enrichment Analysis of DEGs in Responses to K^+^ Treatments

**Figure 4:**
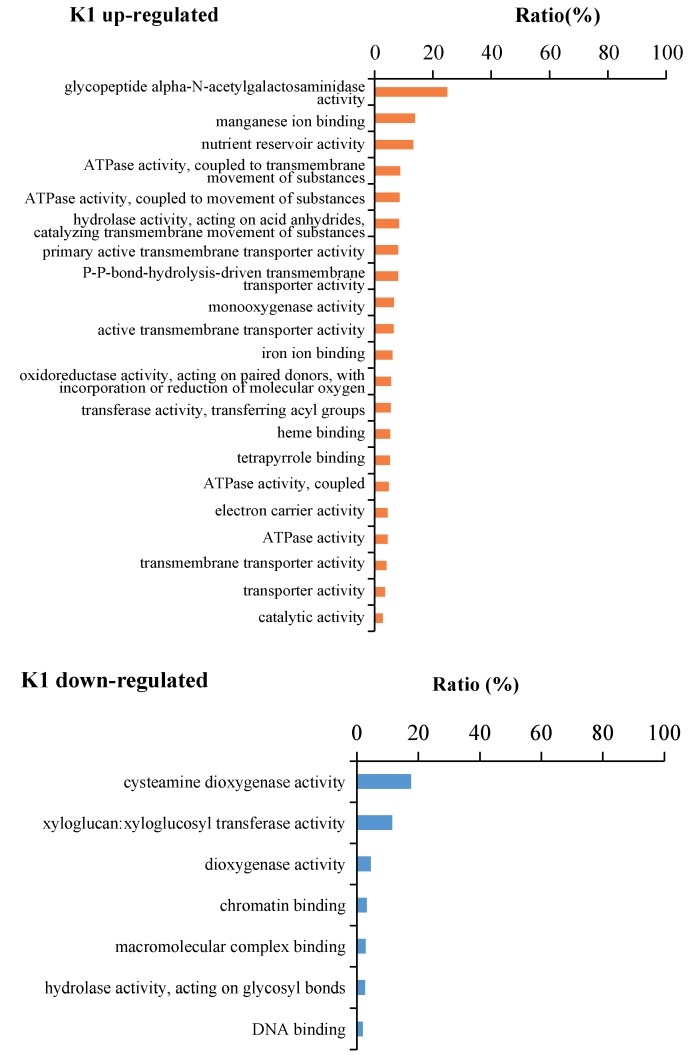

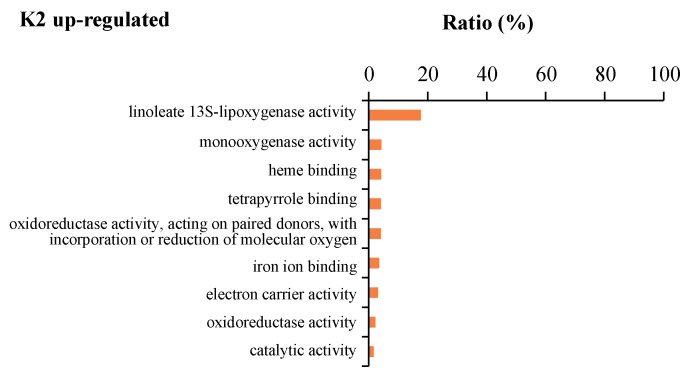

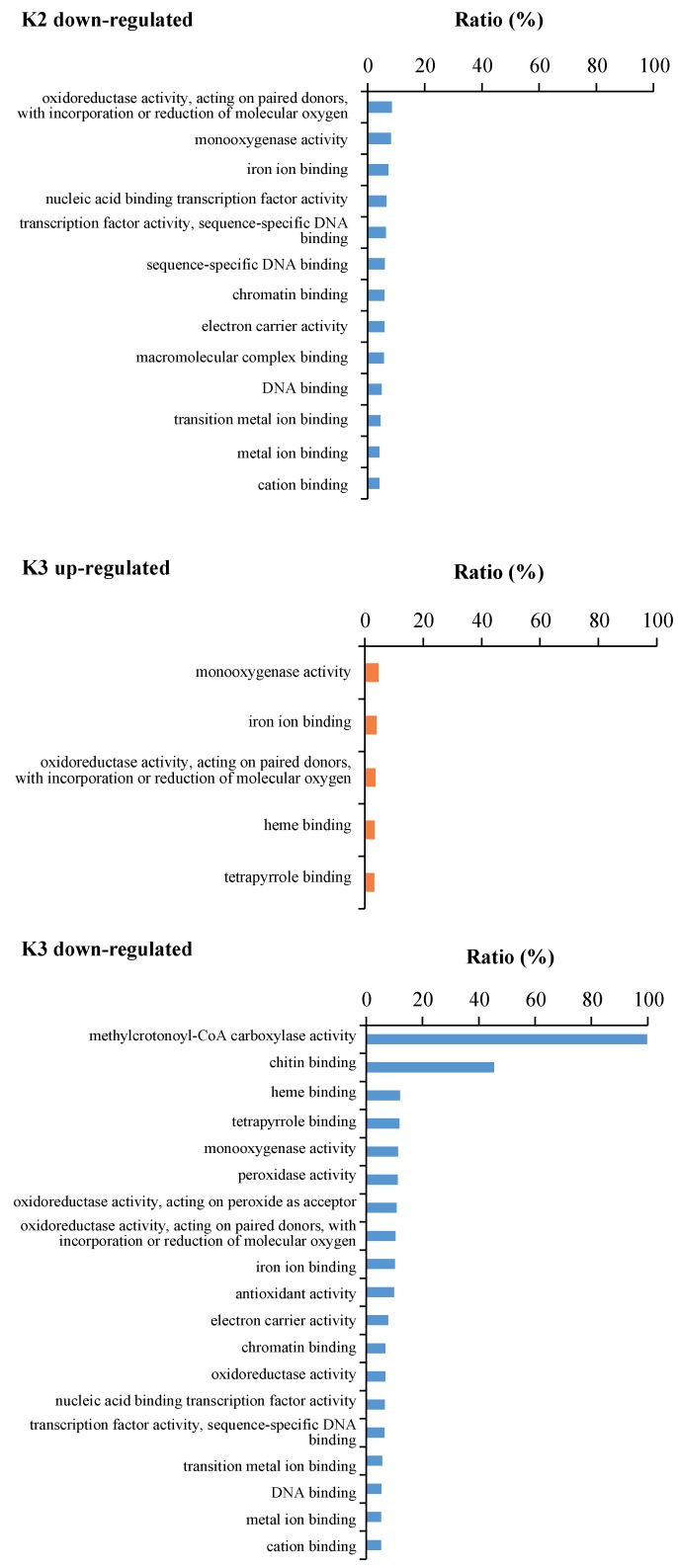
GO molecular function analysis of DEGs detected in banana roots in response to different concentration of potassium. The ratio was the proportion of the number of DEGs in the total number of genes in each GO term. K1 up-regulated: The GO terms significantly enriched in K1 up-regulated DEGs. K1 down-regulated: The GO terms significantly enriched in K1 down-regulated DEGs. K2 up-regulated: The GO terms significantly enriched in K2 up-regulated DEGs. K2 down-regulated: The GO terms significantly enriched in K2 down-regulated DEGs. K3 up-regulated: The GO terms significantly enriched in K3 up-regulated DEGs. K3 down-regulated: The GO terms significantly enriched in K3 down-regulated DEGs.

Gene ontology (GO) analysis was conducted to elucidate the molecular function of the DEGs detected in banana roots from K1, K2 and K3 compared to K0 using the OMICSHARE online program (Table S2). Many GO terms were significantly enriched (adjusted *p* < 0.05) and the GO terms enriched in three kind potassium treatments were different (Table S2). 21 GO terms were enriched in K1 up-regulated DEGs, while 7 GO terms were significantly enriched in K1 down-regulated DEGs. 9 GO terms in K2 up-regulated DEGs, while 13 GO terms were enriched in K2 down-regulated DEGs. 5 GO terms were enriched in K3 up-regulated DEGs, while 19 GO terms were enriched in K3 down-regulated DEGs. In K1 up-regulated DEGs, the “glycopeptide alpha-N-acetylgalactosaminidase activity”, “manganese ion binding”, ”nutrient reservoir activity“, “transporter activity” related, “ATPase activity” related, “hydrolase activity, acting on acid anhydrides, catalyzing transmembrane movement of substances” related, and “transferase activity, transferring acyl groups” GO terms were specific enriched. In K1 down-regulated DEGs, the “cysteamine dioxygenase activity”, “xyloglucan:xyloglucosyl transferase activity”, “dioxygenase activity”, and “hydrolase activity, acting on glycosyl bonds” GO terms were specific enriched In K2 up-regulated DEGs, the “linoleate 13S-lipoxygenase activity” GO term was specific enriched. In K2 down regulated DEGs, the “sequence-specific DNA binding” GO term was specific enriched. In K3 down-regulated DEGs, the “methylcrotonoyl-CoA carboxylase activity”, “chitin binding”, “peroxidase activity”, “oxidoreductase activity, acting on peroxide as acceptor”, and “antioxidant activity” GO terms were specific enriched. While in K3 up-regulated DEGs, no GO term was specific enriched (Figure 4, Table S2). These result suggested that banana roots require genes with different functions to survive with different potassium concentrations.

### 2.5. Expression Trend Analysis

**Figure 5:**
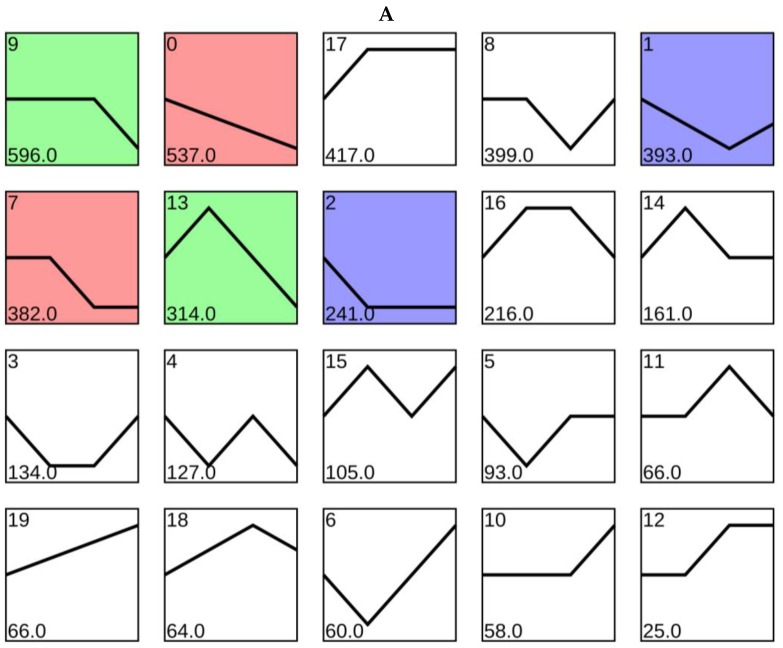

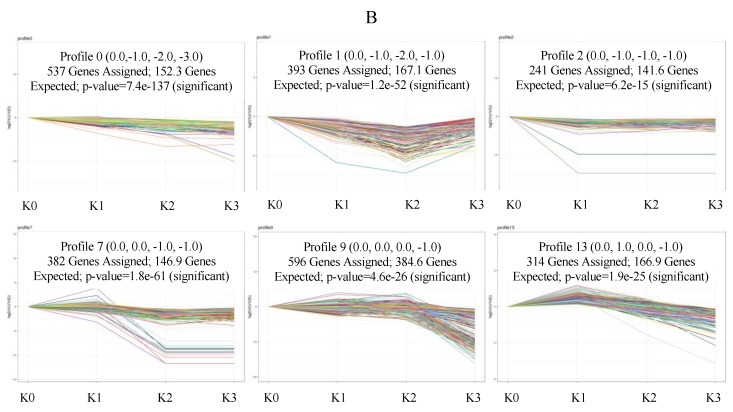
Short time-series expression miner (STEM) clustering on DEGs with the increase of potassium concentration. (**A**), all 20 profiles, with the colored profiles were significant (*p* < 0.05). (**B**), six significant gene expression profiles, with the number of genes and the *p*-value were shown.

The expression profiles of 4454 DEGs were determined and 20 candidate profiles were obtained (Figure 5A), in which six significant (*p* < 0.05) expression profiles (profile 0, 1, 2, 7, 9 and 13) were identified. The 537 DEGs in Profile 0 were significantly present down-regulated with the potassium concentration increase. Profile 1 included 393 DEGs that were down-regulated from K0 to K2 and up-regulated from K2 to K3, which is consistent with the phenotype of the banana root growth. Profile 2 contained 241 DEGs down-regulated from K0 to K1 and present no change from K1 to K3. The 382 DEGs in Profile 7 presented no change from K0 and K1, K2 and K3, but down regulated from K1 to K2. In profile 9, the 596 DEGs present no change in K0 to K2, but down-regulated from K2 to K3. In profile 13, 314 DEGs up-regulated from K0 to K1, then down-regulated to K3 (Figure 5).

### 2.6. GO Enrichment Analysis of the Six Significant Gene Expression Patterns

To understand the functional enrichment of DEGs in the six significant profiles, we conducted GO analysis on them respectively, and regarded the adjusted *p* value < 0.05 as significant enrichment. There were 4, 1, 19, 21, and 2 GO terms significantly enriched in profiles 0, 1, 7, 9 and 13, and zero enriched in profile 2. Significant GO terms that were related to ion binding such as iron ion binding, transition metal ion binding, and manganese ion binding were enriched in profiles 7, 9, and 13. Significant GO terms such as transcription factor activity were enriched in profiles 0 and 7; oxidoreductase activity related GO terms were enriched in profiles 1, 7, and 9 (Table 2). These results suggest that the banana root has evolved a range of molecular strategies that response in different potassium concentrations.

### 2.7. Interaction Network Construction of the DEGs in Significant Expression Patterns

**Figure 6:**
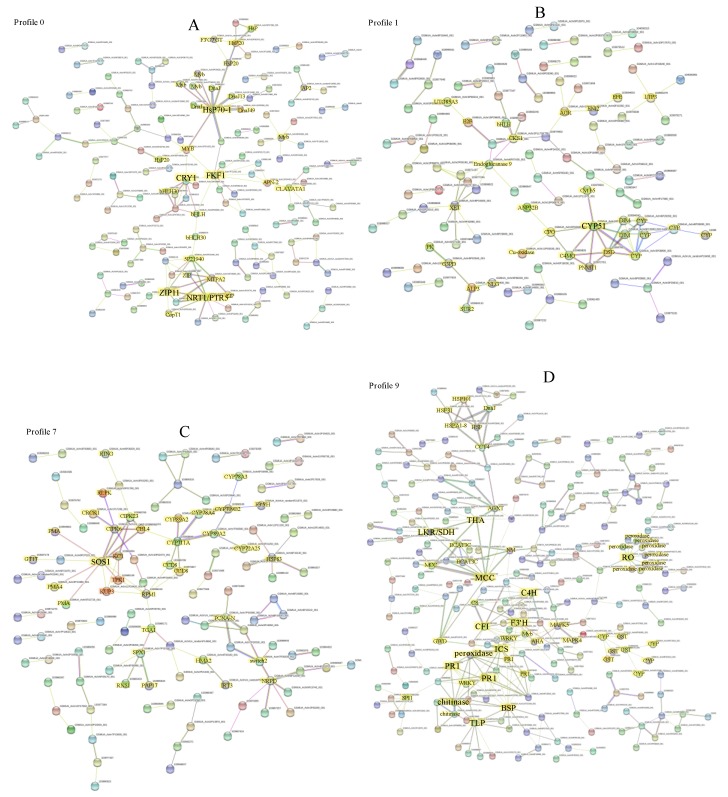
Interaction network of DEGs in profile 0, 1, 7 and 9. The hub genes were represented in the largest font. (**A**) Interaction network of DEGs in profile 0. (**B**) Interaction network of DEGs in profile 1. (**C**) Interaction network of DEGs in profile 7. (**D**) Interaction network of DEGs in profile 9.

To further reveal the interactive relationship among DEGs in the above six significant profiles, the DEGs in each pattern were uploaded to the STRING database to get the interaction network, respectively. In the profile 0 interaction network, five genes, including *FKF1* (flavin-binding kelch repeat F-box protein 1, GSMUA_Achr7P11200_001), *HsP70-1* (Heat shock cognate 70 kDa protein, GSMUA_Achr6P34210_001), *NRT1/PTR5* (Peptide transporter PTR5, GSMUA_Achr5P22130_001), *CRY1* (Cryptochrome-1, GSMUA_Achr6P02100_001), and *ZIP11* (Zinc transporter 11, GSMUA_Achr8P26320_001), were strongly associated with other genes (connected notes more than 8), indicated that they were the core genes in profile 0 (Figure 6A). The five hub genes were connected by several transcription factors including *MYBs* and *bHLHs*. There were also many *HsP* transcription factors in the network (Figure 6A, Tables S3 and S4). In the profile 1, *CYP51* (Sterol 14-alpha-demethylase/Obtusufoliol 14-demethylase, Cytochrome P450 family, GSMUA_Achr10P12080_001), was the hub gene with the highest node degree 8 (Figure 6B, Tables S5 and S6). It direct interacted with one another *CYP* members, two *DIMs* (Cell elongation protein DIMINUTO), *PNMT1* (Phosphoethanolamine N-methyltransferase 1), two sterol desaturase family members (*C4MO*, C-4 methylsterol oxidase; DSD, Delta(7)-sterol-C5(6)-desaturase), and one *CPO* (Coproporphyrinogen-III oxidase). In profile 7, *SOS1* (Sodium/hydrogen exchanger 7, GSMUA_Achr8P16670_001) was the hub gene in the PPI network, exhibiting the highest (10) node degree (Figure 6C, Tables S7 and S8). Potassium transport-related genes such as *KCO1*, *KT4*, and *KAT3*, were direct or indirect connected with *SOS1*. Calcineurin B-like protein 4 (CBL), Disease resistance protein RPM1, CBL-interacting protein kinases (CIPK*6* and *CIPK23*), and 3 Plasma membrane ATPase (*PMA*) family members were direct connected with *SOS1*. The *SOS1* also indirect interacted with several *CYP* family members through *CBL* (Figure 6C). In profile 9, *THA* (Putative L-allo-threonine aldolase, GSMUA_Achr2P12200_001), *LKR/SDH* (Alpha-aminoadipic semialdehyde synthase, GSMUA_Achr10P23170_001), *MCC* (Methylcrotonoyl-CoA carboxylase beta chain, GSMUA_Achr1P21350_001), *C4H* (Trans-cinnamate 4-monooxygenase, GSMUA_Achr7P18320_001), *CFI* (Chalcone-flavonone isomerase, GSMUA_Achr4P16830_001), *F3′H* (Flavonoid 3′-monooxygenase, GSMUA_Achr3P30350_001), *PR1s* (Pathogenesis-related protein 1, GSMUA_Achr4P23100_001, GSMUA_Achr2P13240_001), *BSP* (Secretory protein, GSMUA_Achr11P22160_001), *TLP* (Thaumatin-like protein, GSMUA_Achr6P31470_001), *ICS* (Isochorismate synthase, GSMUA_Achr3P32520_001), *RO* (Reticuline oxidase, GSMUA_Achr3P23450_001), chitinase (GSMUA_Achr3P26900_001), and peroxidase (GSMUA_Achr9P23850_001), were the hub genes with the node degree more than 8 (Figure 6D, Tables S9 and S10). In profile 2 and profile 13, there were no gene with the node degree more than 8 (Figures S1 and S2). In profile 2, F-box/LRR-repeat protein 3 (GSMUA_Achr10P29380_001) was the hub gene with 4 nodes (Figure S1, Tables S11 and S12). In profile 13, *MS* (Malate synthasewas, GSMUA_Achr8P28810_001) was the hub gene and exhibited 7 nodes (Figure S2, Tables S13 and S14).

### 2.8. Validation of RNA Sequencing Data

**Figure 7:**
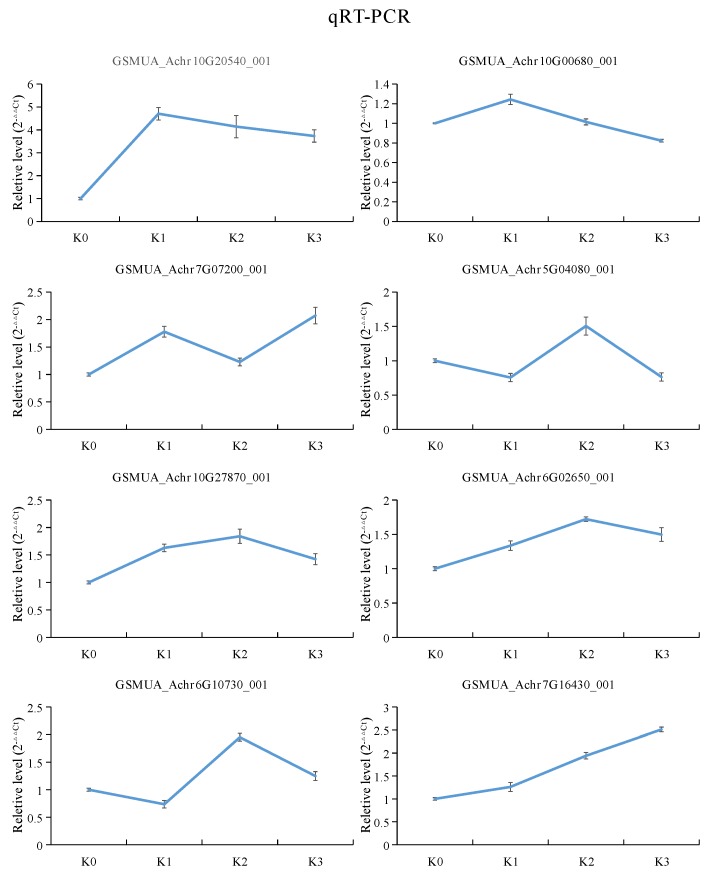
Relative gene expression of 8 randomly selected genes. The blue lines represent the relative intensity of real-time quantitative RT-PCR. The experiments were repeated three times. The error bars represent ± SE. *n* = 3.

Quantitative real-time PCR (qRT-PCR) were performed to evaluate the accuracy of the gene expression in this transcriptome data. In this study, eight DEGs were randomly selected to detected by qRT-PCR. The expression patterns analysis showed that the qRT-PCR results were consistent with the transcriptome patterns. This result indicated that the RNA-Seq data was reliable (Figure 7).

## 3. Discussion

Plant roots can sense changes in the external environment and adjust the absorptive capacity by changing root morphology [30]. The length and surface area of a root system can be enhanced in potassium-rich soil, while the length and surface area of root system can be restricted in potassium-deficient soil [31,32,33]. Thus, it was not surprising to detect the inhibition of banana root hair growth in potassium deficiency (K0) and low potassium (K1) treatments, in the present study. As it was rarely reported the regulation of high potassium levels on plant root growth, it was meaningful to find out that the banana root length increased and root hairs were inhibited under the high potassium (K3) treatment. With the purpose to finding out the mechanism of different potassium levels in regulating the banana root growth, we performed the RNA-seq transcriptome analysis. The expression profile analysis of DEGs showed six significantly profiles, including profile 0, 1, 2, 7, 9 and 13 (Figure 6). Coincidentally, the expression trend of 393 DEGs in profile 1 was contrary with the root growth phenotype change trend treated by different levels of potassium. These genes were up-regulated by potassium stresses compared with normal potassium level, and it is speculated that they were the main genes leading the change of root phenotype. GO enrichment analysis showed that one GO term (including 17 DEGs), which is related to oxidoreductase activity, was significantly enriched in profile 1 (Table 2). The 17 DEGs included nine Cytochrome P450 family members, four oxidoreductase family members, two 1-aminocyclopropane-1-carboxylate oxidase family members, one Omega-6 fatty acid desaturase, and one S-norcoclaurine synthase (Table S15). The oxidoreductase activity enrichment also had reported in other plants treated by low nutrient stresses, such as in low potassium treated pear fruit [34], in low nitrogen treated wheat [35], in low phosphorus treated sorghum [36]. The networks of the DEGs in profile 1 were conducted, and the results revealed that *CYP51*, a member of cytochrome P450 family was the hub gene with the highest node degree 8 (Figure 6B, Tables S5 and S6). In low potassium treated pear fruit, there multiple cytochrome P450 members were detected [34]. In low potassium treated tobacco, 3 cytochrome P450 members were also up-regulated [37]. The DEGs belong to cytochrome P450 family were also detected from potassium deficiency tomato [38], low-N polar [39], rice [40], sorghum [41] and wild barley [42]. Cytochrome P450 family genes were reported to participate in the biosynthesis of lignin precursors or hormone homeostasis [43], involved in the formation of cell wall and regulated the plant growth [44]. Therefore, the focus of *CYP51* gene in DEGs interaction network of profile 1, indicated a significant correlation with root morphological changes under potassium stresses.

In profile 7, the DEGs were high regulated at low potassium levels (K0, K1) compared to K2 and K3 (Figure 6). GO analysis showed that the ion binding, transporter activity, Kinase, oxidoreductase activity, Transcription factors related DEGs were significantly enriched in profile 7 (Table 2). The DEGs interaction network indicated that *SOS1* was the hub gene with 10 nodes (Figure 6C, Tables S7 and S8). *SOS1* is a plasma membrane Na^+^/H^+^ antiporter and belongs to the “salt overly sensitive” (SOS) signaling pathway [45]. It was reported to be an important gene locus for salt tolerance and potassium acquisition in plants [46,47,48]. Previous studies showed that *SOS1* plays an essential function in regulating the spatial distribution and long-distance transportation of Na^+^ and K^+^, the transport system of K^+^ and Na^+^, keeping the Na^+^ and K^+^ homeostasis, and maintaining the normal growth of plants [49]. Here, the *SOS1* was at a pivotal position in the interaction network of profile 7 DEGs, indicating that it played a key role in regulating banana root response to potassium stress. Early studies revealed that the *CBL1/CBL9-CIPK23-AKT1* module is the primary K^+^ uptake regulatory pathway to plants response in potassium starvation [50]. However, in our study, the *AKT1, CBL1*, and *CBL9* were not present in the interacting networks of profile 7. Then we dug into the transcriptome data and found that they were also not present in 4454 DEGs. The results suggested that the *CBL1/CBL9-CIPK23-AKT1* pathway was not involved in banana root system in response to potassium stress. *AtKC1* is a K^+^ channel regulatory subunit, which together with *AtCIPK23*, has been reported to coordinate the regulation of *AtAKT1*-mediated K^+^ short stress response in *Arabidopsis* [51,52]. In addition, the *CBL4/CIPK6* complex was revealed to modulating *AKT2* activity in *Arabidopsis* [53]. *CBL4* (*SOS3*) interacted with *SOS2*, and further activated *SOS1* by phosphorylation [54,55,56]. *TPK1*, a voltage independent, Ca^2+^/pH-activated two-pore K^+^ channel, was proved playing key functions in keeping intracellular K^+^ homeostasis [57,58]. *KUP3* belongs to the largest potassium transporter *HAK/KUP/KT* family, which was strong inducted by potassium starvation [59]. In this study, it was predicted that *KC1* interacted with *CIPK23, TPK1* and *KUP3* respectively, and *CIPK23* also interacted with *SOS1* and *CBL4*(*SOS3*) respectively (Figure 6C). Given that the interaction between *SOS1* and *SOS3* [56], *KC1* and *CIPK23* [51,52], *CBL4*(*SOS3*) and *CIPK6* [53] has been proved, thus the consistent expression and their interaction of *SOS1, KC1*, *CIPK23, CBL4, CIPK6, KUP3, TPK1* in the network, provide new possible mechanisms for banana roots to response to potassium stress. In a word, the genes in profile 7 were mainly related to ion transport of potassium.

The 537 DEGs in Profile 0 were significantly down-regulated with the potassium concentration increase. In the profile 0 PPI network, five genes, including *FKF1*, *HsP70-1, NRT1/PTR5, CRY1*, and *ZIP11*, were strongly connected to other genes, indicated that they were hub genes in profile 0 (Figure 6A). The five hub genes were connected by several transcription factors including MYBs and *bHLHs*. There were also many *HsP* transcription factors in the network (Figure 6A, Tables S3 and S4). A recent study showed that cryptochrome 1 (*CRY1*, plant blue light photoreceptor) actively participates in the formation of secondary cell walls. When *CRY1* gene was missing in plants, it will lead to the decrease of cellulose and lignin accumulation in plant stems [60]. Later, FLAVIN-BINDING KELCH REPEAT, F-BOX 1 (*FKF1*), another blue light receptor, was identified from cellulose synthase-deficient dwarf mutant, and revealed its role in negatively regulation of cellulose biosynthesis [61]. In this study, the hub location of *CRY1* and *FKF1* in the interaction network of profile 0, indicated that the banana roots might change their cell wall component to response to different potassium stress. In this network, ZIP11, a zinc transporter gene, existed in the hub location. Members of the ZIP family were considered to be important transporters of Zn, Fe, Mn, Cu and other bivalent metal ions [62,63,64]. Previous studies indicated that the relationship between Zinc and potassium in plants is mutual promotion. As the content of Zn in the body increases, the content of K also increases in cotton [65]. *ZIP11* has the function of separating Zn/Cd into vesicles and holding back its transfer from root to bud [66]. In addition, there were also some other *ZIP* or metal transport related genes such as *IRT1, HMA2, ZIP5* in this interaction network. Therefore, the different potassium stress in banana roots might cause different zinc or other metal levels in roots through the ZIPs regulating network. A *NRT1/PTR* family gene, known for specific nitrate and auxin uptake, was found in the hub location of the interaction network of profile 0. It is well known that the nitrogen and potassium fertilizers are interdependent. The expression of several *NRT1/PTR* genes regulated by external potassium has also been reported on tomato [67], and Arabidopsis [16]. In addition, it has been mentioned that NRT1.1 plays a key role in the K^+^ translocation of *Arabidopsis thaliana* [68]. The present results were consistent with previous studies, indicating that the exogenous potassium stress can also affect the absorption and transport of nitrogen in banana root. Plant heat shock protein (*Hsps*) is a kind of protein that can be activated and produced in large quantities when plants encounter abiotic stress. Hsps has the function of molecular chaperone. It can maintain the stability of protein and cell membrane by assisting protein folding, assembly, translocation and degradation, and protect the normal growth of plants [69]. The *HSP70* expression has been paid attention in plant response to abiotic stress, for instance drought [70,71] and salt [35,72]. However, its expression in plants response to potassium stress, was rarely mentioned. Nevertheless, its role in human potassium channels has been proven [72,73]. According to the above analysis, genes in profile 0 were mainly related to regulating cell wall formation, other nutrition transport and the Hsps expression.

In profile 9, the 596 DEGs present no change from K0 to K2, but down-regulated from K2 to K3. In profile 9, *THA, LKR/SDH, MCC, C4H, CHI, F3′H, PR1s, BSP, TLP, ICS, RO*, chitinase, peroxidase were the hub genes with the node degree more than 8 (Figure 6D, Tables S9 and S10). *THA, LKR/SDH, MCC, C4H, CHI, F3′H, ICS*, chitinase and peroxidase belong to the metabolic pathways. Threonine aldolase (*THA*) is involved in the metabolic process of threonine. Threonine is lysed into glycine and acetic acid under the action of threonine aldolase. The results showed that glycine, under the action of glycine methyltransferase, synthesized betaine through three steps of methylation, thus improving the stress resistance of plants [74]. *THA* may be an important step in the regulation of amino acid metabolism in banana roots under adverse potassium conditions. *LKR/SDH* (Lysine ketoglutaric acid reductase/saccharopine dehydropine dehydrogenase) participating in the Lys degradation, is found to be a bifunctional enzyme [75]. The expression of *LKR/SDH* was affected by lysine, osmotic stress, abscisic acid, sugar, jasmonate, nitrogen, salt and drought stresses [76,77,78]. This study indicated that, the expression of *LKR/SDH* was depressed by high concentration of potassium. Leucine is a branched amino acid involved in the breakdown of large amounts of protein, which accumulates instantaneously in carbon-hungry cells. Methylcrotonoyl-CoA carboxylase (*MCC*), a mitochondria-localized carboxylase, is involved in the leucine catabolizing pathway [79,80,81]. It is expressed in a large number of sycamore cells during starvation [81,82]. Therefore, MCCase has been proposed as a new biochemical marker of carbohydrate starvation autophagy process [79]. Knockout of *MCCa* or *MCCb* results in impaired reproductive growth phenotypes in *Arabidopsis thaliana* [82]. It was also reported that *MCC* plays an important role in regulating triglyceride accumulation in *Phaeodactylum tricornutum* [83]. During cotyledon development, MCCase mRNA accumulation was positively correlated with the increase of organ age [80]. Biotinylation, a post-translational modification, is important for the activity of *MCC*, which has been demonstrated in tomato [84]. The expression profile of *MCC* in banana roots treated by different potassium levels, which provided new evidence for its molecular regulatory function. Cinnamate 4-hydroxylase (C4H, EC 1.14.13.11) is one of the key enzymes in the phenylpropanoid pathway, which is also the first Cyt P450-dependent monooxygenase of the pathway [85]. The expression of *C4H* can be regulated by various environmental stresses. In *Arabidopsis* [86], *Dryopteris fragrans* [87], and *Salvia miltiorrhiza* [88], *C4H* was induced by light, UVB. In rice [89], orange [90], *Carthamus tinctorius* [91] and kenafcold [92], *C4H* was induced by NaCl, cold, H_2_O_2_, ABA and salicylic acid (SA). In Capsicum [93], C4H was induced by drought stress. Chalcone isomerase (*CHI*; EC 5.5.1.6) catalyze conversion of chalcone to flavonoids to produce most major flavonoid subgroups [94]. The correlation between CHI activity and plant response to environmental stress has been investigated. For example, the addition of fungal inducers led to rapid accumulation of *CHI* mRNA in bean cell culture [95]; overexpressing *GmCHI1A* can decrease the harm by *Phytophthora sojae* in soybean [96]. Similarly, the transcription of *CHI* gene was induced by salt stress [97,98,99]. F3′H belongs to the cytochrome P450 subfamily and catalyzes the NADPH-dependent monooxygenase reactions [100]. It also participates in the flavonoid biosynthetic pathway. Our work showed that *C4H, CHI*, and *F3′H* in banana root were significantly down-regulated under high level potassium stress. In addition, they were also in the hub of the interaction network. It indicates that high potassium stress may inhibit the flavonoid biosynthesis pathway dominated by *C4H, CHI* and *F3′H* genes in banana roots. Peroxidase (POD, EC 1.11.1.7) is a recognized enzyme that affects plant growth through regulation of the lignin accumulation [101,102]. The increase of ion-binding POD activity inhibited root growth of rice [103]. By adding substances that inhibit pod activity, cell wall rigidity caused by pod can be removed and root elongation can be promoted [104]. Therefore, the down-regulation of peroxidase gene expression in profile 9 under high potassium treatment, might be the main reason which cause the banana root length increase. It is well known that *PR1*, chitinases, thaumatin-like proteins, peroxidases have been classified as PR proteins, which are associated with plant defense [105]. Isochorismate synthase 1 (*ICS1*) is an essential enzyme in the synthesis of salicylic acid (SA). The SA can be synthesized by isochorismate synthase (ICS) pathway, and also can be synthesized by phenylalanine ammonia-lyase (PAL) pathway in plants [106,107]. In Arabidopsis, PAL pathway is mainly for the basic production of SA, while ICS pathway is for the production of pathogen-induced SA [108,109,110]. The accumulation of SA can induce the expression of the pathogenesis-related gene (*PR1*), which is considered to be an SA receptor in plants [111,112,113,114]. Our study showed that the chitinases, thaumatin-like proteins, peroxidases, *ICS1*, and 2 *PR1* genes were down regulated under high level potassium stress. As they were located in the core location, therefore, it was speculated that high concentration of potassium inhibited their expression and would reduce the disease resistance of banana roots. In conclusion, the genes in profile 9 were suppressed by high potassium, and these genes mainly involved in the metabolic function and plant defense.

## 4. Materials and Methods

### 4.1. Plant Materials and Different Potassium Treatments

One-month-old young Brazilian bananas were washed and transplanted into plastic containers filled with vermiculite and grown in a greenhouse. The culture condition was 28 ± 1 °C, 16 h/d light, 60–70% relative humidity. The plants were wet thoroughly by special nutrient solution every 5 days. Basic nutrient solution was made up of 4 mmol/L Ca(NO_3_)_2_·4H_2_O, 3 mmol/L NH_4_NO_3_, 1 mmol/L NH_4_H_2_PO_4_, 2 mmol/L MgSO_4_·7H_2_O, iron salts and micronutrient were formulated with Hoagland nutrient solution. Four potassium levels from no potassium to high potassium were set to add 0 (K0), 0.03 (K1), 3 (K2), 200 (K3) mmol/L K_2_SO_4_ respectively. After four weeks of potassium stress, banana roots were observed, photographed and sampled. The potassium stress treatment of banana root with different concentration was repeated three times.

### 4.2. Extraction of Total RNA from Banana Root under Different Potassium Stresses

Total RNA of banana root treated with different potassium was extracted according to the instructions of Trizol Kit (Promega, USA), after that the RNase-free DNase I (Takara Bio, Shiga, Japan) was added to eliminate residual DNA. The 2100 Bioanalyzer (Agilent Technologies, Santa Clara, CA, USA) was used to checked the RNA quality and concentration. Equal amounts of RNA from each sampled tissue were mixed for the subsequent steps of our experiments. Three biological replicates were performed and each biological replicate was constituted by a pool of three plants.

### 4.3. RNA-Seq Library Preparation, Sequencing and Data Analysis

The construction method of the library was the same as that reported by Balic et al. [115]. After being purified and enriched, the 200 bp ± 25 bp cDNA fragments were used to construct cDNA library. The cDNA library sequencing was performed by Gene Denovo Biotechnology Co. (Guangzhou, China) using the Illumina HiSeq™ 2000. Then the obtained sequences were checked to delete the low-quality sequences. In addition, the retained high-quality sequences were assembled. Sequencing reads were mapped to banana genome sequences (https://banana-genome-hub.southgreen.fr/) by SOAPaligner/soap2. The uniquely mapped reads were used to evaluate the expression level. The formula of RPKM= 106C/NL/103 was used to evaluate the expression level as described before [116]. The RPKM value is then used for pairwise comparisons between samples. The gene expression meeting the condition of *p*-value < 0.05, log2FC ≥ 1 were determined to be significantly different expression. The raw data was uploaded to NCBI, and the BioProject ID is PRJNA589855.

### 4.4. Data Statistics, GO Enrichment, Expression Trend Analysis and Interactive Network Construction of DEGs

The bar chart is drawn on an EXCEL spreadsheet. Analysis of Venn, upset Venn, GO enrichment and gene expression trend were drawn by appropriate software from OMICSHARE platform (https://www.omicshare.com/), respectively. The protein interactive networks were constructed by the STRING online tool (https://string-db.org/).

### 4.5. RNA-Seq Reliability Verification

To assess the accuracy of sequencing data, we randomly selected 8 banana DEGs and the banana actin gene (an internal control), to do the following Quantitative Real-Time PCR analysis (qRT-PCR). Gene-specific primers (Table S16) were designed and analyzed on the online primer tool (https://www.ncbi.nlm.nih.gov/tools/primer-blast/). RT reagent Kit No. RR047Q (TaKaRa, Beijing, China) were used to reverse transcribe RNA into cDNA. The reaction solution of qRT-PCR was configured by the reagent No. RR820L (TaKaRa, Beijing, China). The reaction equipment is ABI 7900 HT Fast Real-Time PCR System (Applied Biosystems, Foster City, CA, USA). The relative gene expression value was analyzed using the 2^−ΔΔCt^ formula, and three biological replicates were used in this research. The data was statistically analyzed and figured by Excel.

## 5. Conclusions

In this study, the morphological changes and DEGs of banana roots under different potassium stress were systematically understood. The results suggested that different potassium concentrations had various effects on the root phenotype of banana. 4454 DEGs were obtained in roots. Gene function classification enrichment was shown in the paper. six significant expression profiles were conducted. The core genes in each expression pattern were screened out. In conclusion, this study revealed the influence of potassium level on banana root system and the molecular regulation mechanism and provided a new idea for improving the use efficiency of banana potassium.

## Figures and Tables

**Table 1:** Summary of RNA-seq data and reads mapping.

	Raw Data	Clean Data	Q20 (%)	GC (%)	Unique Mapped	Multiple Mapped	Mapping Ratio (%)
K0	28,043,505	27,574,822	94.01	53.96	21,674,956	92,502	79.23
K1	30,165,251	29,675,721	94.13	53.58	22,955,324	74,451	77.27
K2	35,061,437	34,465,589	94.02	53.95	27,718,870	94,409	79.81
K3	24,438,772	24,024,081	94.15	54.26	20,030,295	50,940	83.37

**Table 2:** Summary of GO molecular function analysis of six profiles.

	GO Terms	Description	Out	All	*p*.Adjust	Ratio (%)
Profile 0	GO:0003682	chromatin binding	23	585	0.007589488	3.93
	GO:0003700	transcription factor activity, sequence-specific DNA binding	35	1046	0.006265828	3.35
	GO:0001071	nucleic acid binding transcription factor activity	35	1057	0.006265828	3.31
	GO:0003677	DNA binding	77	2659	0.00010964	2.90
Profile 1	GO:0016705	oxidoreductase activity, acting on paired donors, with incorporation or reduction of molecular oxygen	17	460	0.007235669	3.70
Profile 7	GO:0008131	primary amine oxidase activity	3	10	0.01050813	30.00
	GO:0016641	oxidoreductase activity, acting on the CH-NH2 group of donors, oxygen as acceptor	3	14	0.013873365	21.43
	GO:0048038	quinone binding	4	25	0.01050813	16.00
	GO:0016638	oxidoreductase activity, acting on the CH-NH2 group of donors	3	24	0.04293938	12.50
	GO:0004713	protein tyrosine kinase activity	4	50	0.044647033	8.00
	GO:0004497	monooxygenase activity	11	255	0.012179367	4.31
	GO:0016705	oxidoreductase activity, acting on paired donors, with incorporation or reduction of molecular oxygen	16	460	0.01050813	3.48
	GO:0008324	cation transmembrane transporter activity	15	434	0.012179367	3.46
	GO:0009055	electron carrier activity	17	506	0.01050813	3.36
	GO:0005506	iron ion binding	13	420	0.040286999	3.10
	GO:0003700	transcription factor activity, sequence-specific DNA binding	32	1046	0.000516422	3.06
	GO:0001071	nucleic acid binding transcription factor activity	32	1057	0.000516422	3.03
	GO:0043565	sequence-specific DNA binding	19	644	0.013547559	2.95
	GO:0004674	protein serine/threonine kinase activity	26	1080	0.023435321	2.41
	GO:0004672	protein kinase activity	33	1498	0.023435321	2.20
	GO:0003677	DNA binding	53	2659	0.012179367	1.99
	GO:0046914	transition metal ion binding	39	1957	0.040286999	1.99
	GO:1901363	heterocyclic compound binding	123	7912	0.035080217	1.55
	GO:0097159	organic cyclic compound binding	123	7923	0.035080217	1.55
Profile 9	GO:0004485	methylcrotonoyl-CoA carboxylase activity	2	2	0.010998085	100.00
	GO:0008061	chitin binding	6	11	1.37 × 10^−6^	54.55
	GO:0004084	branched-chain-amino-acid transaminase activity	2	4	0.037470895	50.00
	GO:0052654	L-leucine transaminase activity	2	4	0.037470895	50.00
	GO:0052655	L-valine transaminase activity	2	4	0.037470895	50.00
	GO:0052656	L-isoleucine transaminase activity	2	4	0.037470895	50.00
	GO:0004568	chitinase activity	6	22	8.26 × 10^−5^	27.27
	GO:0008762	UDP-N-acetylmuramate dehydrogenase activity	5	38	0.018018015	13.16
	GO:0004601	peroxidase activity	15	160	1.86 × 10^−5^	9.38
	GO:0016684	oxidoreductase activity, acting on peroxide as acceptor	15	166	2.48 × 10^−5^	9.04
	GO:0016209	antioxidant activity	16	191	2.48 × 10^−5^	8.38
	GO:0020037	heme binding	30	446	1.74 × 10^−7^	6.73
	GO:0046906	tetrapyrrole binding	30	455	1.86 × 10^−7^	6.59
	GO:0050660	flavin adenine dinucleotide binding	9	157	0.043604233	5.73
	GO:0004497	monooxygenase activity	12	255	0.045573268	4.71
	GO:0016705	oxidoreductase activity, acting on paired donors, with incorporation or reduction of molecular oxygen	20	460	0.010998085	4.35
	GO:0004553	hydrolase activity, hydrolyzing O-glycosyl compounds	20	488	0.019555184	4.10
	GO:0005506	iron ion binding	17	420	0.037470895	4.05
	GO:0016491	oxidoreductase activity	69	1744	1.74 × 10^−7^	3.96
	GO:0016798	hydrolase activity, acting on glycosyl bonds	20	516	0.035644596	3.88
	GO:0003824	catalytic activity	224	9724	3.52 × 10^−5^	2.30
Profile 13	GO:0030145	manganese ion binding	5	43	0.01549229	11.63
	GO:0045735	nutrient reservoir activity	5	45	0.01549229	11.11

Note: Ratio = Out/All*100.

## Supplementary Materials

The following are available online at https://www.mdpi.com/2223-7747/9/1/11/s1, Figure S1: Interaction network of DEGs in profile 2, Figure S2: Interaction network of DEGs in profile 13. Table S1: Transcript abundance measurements at each potassium concentration treatment, Table S2: GO molecular function analysis of DEGs detected in banana roots in response to different concentration of potassium, Table S3: annotation of genes in profile 0, Table S4: The interaction data of DEGs in profile 0, Table S5: annotation of genes in profile 1, Table S6: The interaction data of DEGs in profile 1, Table S7: annotation of genes in profile 7, Table S8: The interaction data of DEGs in profile 7, Table S9: annotation of genes in profile 9; Table S10: The interaction data of DEGs in profile 9, Table S11: annotation of genes in profile 2, Table S12: The interaction data of DEGs in profile 2, Table S13: annotation of genes in profile 13, Table S14: The interaction data of DEGs in profile 13, Table S15: Annotation of DEGs of the significant GO term in profile 1, Table S16: Primer information for qRT-PCR.
