# Supplementary material for: Transcriptome Changes Induced by Different Potassium Levels in Banana Roots
Source: Plants (Basel). 2019 Dec 19;9(1):11. doi: 10.3390/plants9010011 (PMC7020221; doi:10.3390/plants9010011)
Supplement: Supplementary file 1 [file plants-09-00011-s001.zip › Supplementary figures.docx]

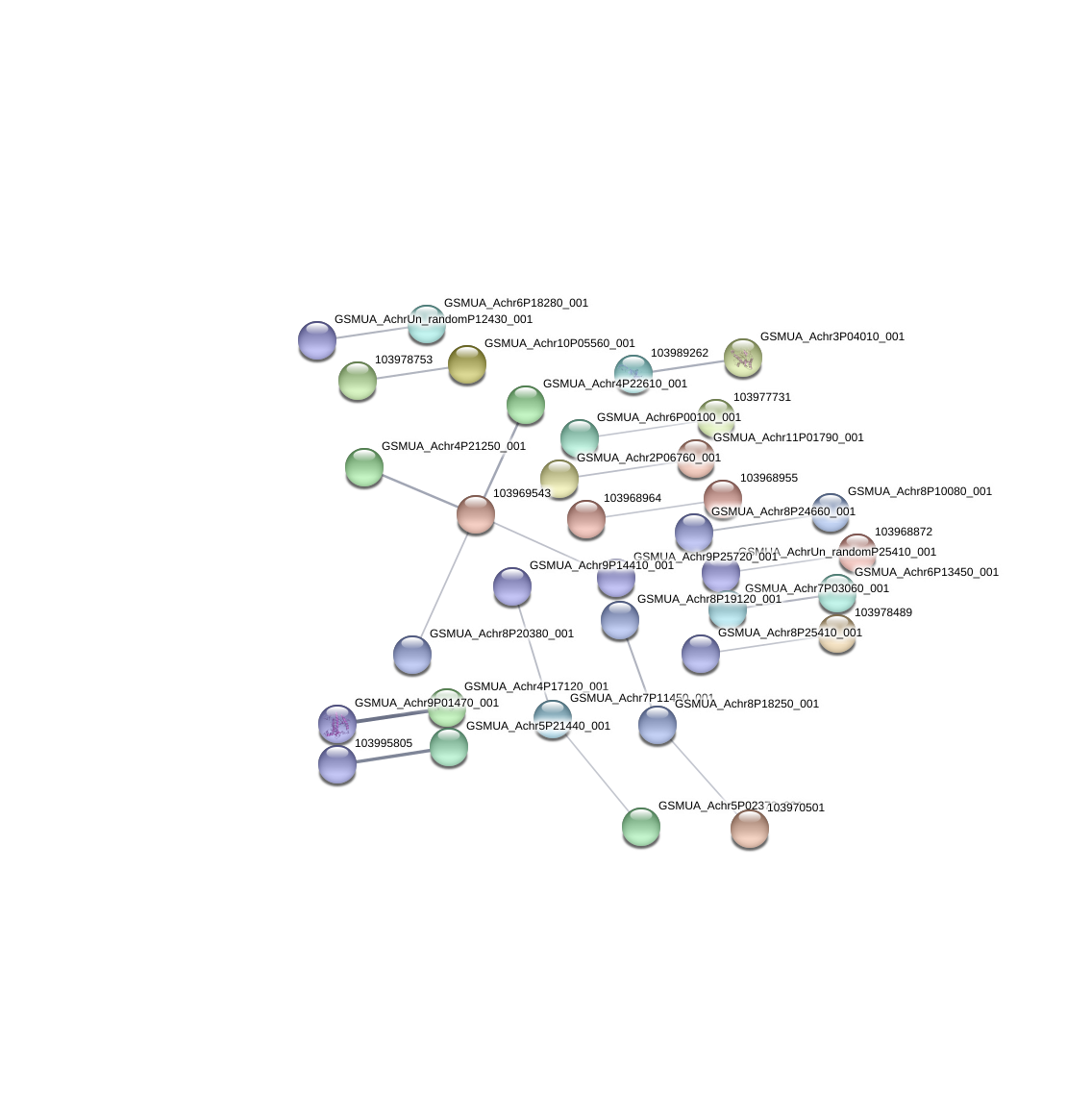


**Figure S1.** Interaction network of DEGs in profile 2.


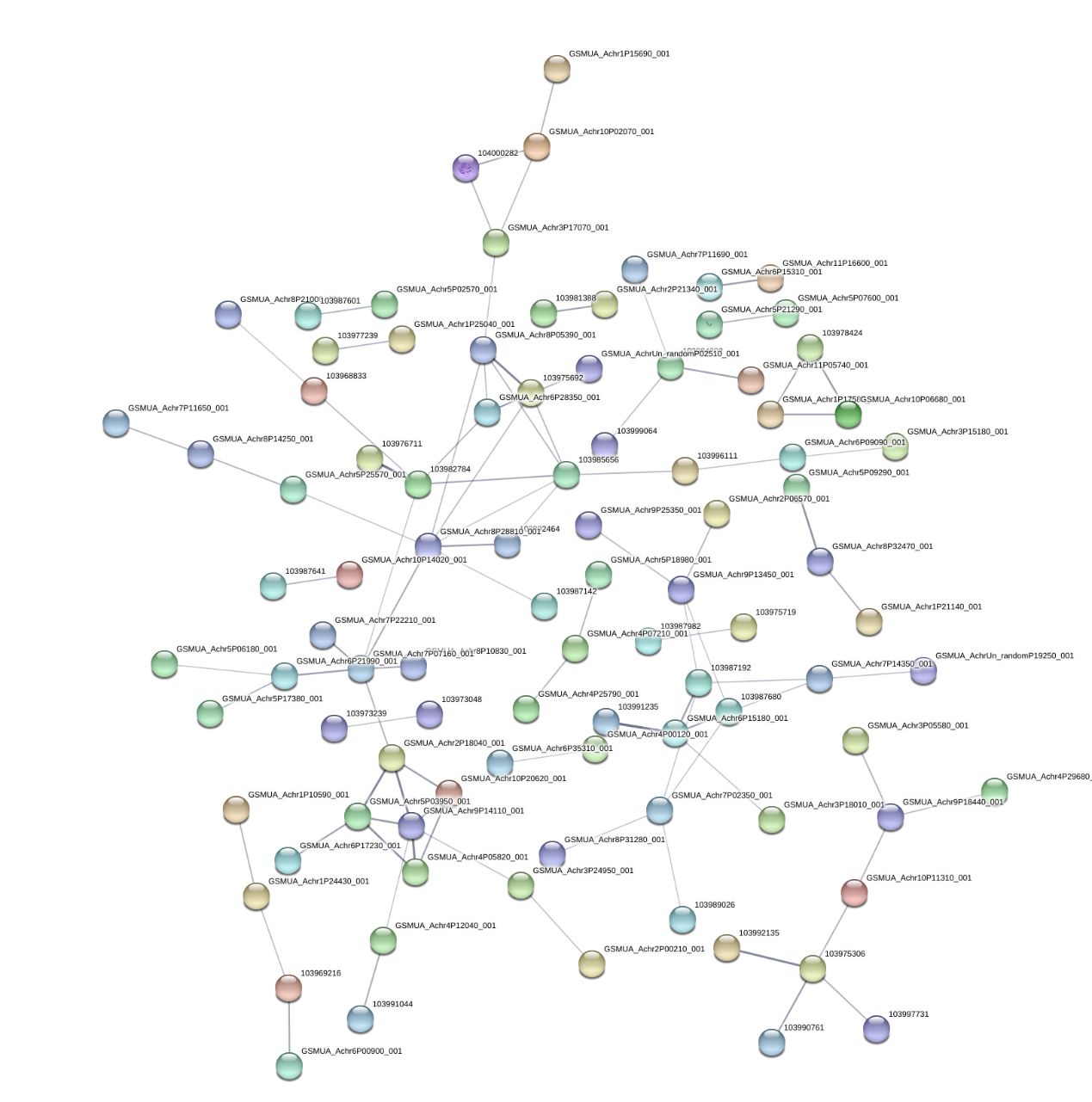


**Figure S2.** Interaction network of DEGs in profile 13.
